# Emerging Role of EGFR Mutations in Creating an Immune Suppressive Tumour Microenvironment

**DOI:** 10.3390/biomedicines10010052

**Published:** 2021-12-27

**Authors:** Simran S. Kapoor, Dietmar M. W. Zaiss

**Affiliations:** 1 Institute of Immunology and Infection Research, University of Edinburgh, Edinburgh EH9 3FL, UK; s1708642@sms.ed.ac.uk; 2 Faculty of Medicine, Institute of Immune Medicine, University of Regensburg, 93053 Regensburg, Germany

**Keywords:** Epidermal Growth Factor Receptor (EGFR), Immune Checkpoint Inhibitors (ICI), tumour immunotherapy, Tumour Microenvironment (TME), lymphocyte depletion, Transforming Growth Factor beta (TGFβ)

## Abstract

Several types of tumours overexpress the Epidermal Growth Factor Receptor (EGFR) in either wild type or mutated form. These tumours are often highly aggressive and difficult to treat. The underlying mechanisms for this phenomenon have remained largely unresolved, but recent publications suggest two independent mechanisms that may contribute. According to one line of research, tumours that overexpress the EGFR grow autonomously and become “addicted” to growth factor signalling. Inhibition of this signal using EGFR inhibitors can, therefore, induce cell death in tumour cells and lead to tumour shrinkage. The other line of research, as highlighted by recent findings, suggests that the overexpression, specifically of mutant forms of the EGFR, may create an immune-suppressive and lymphocyte depleted microenvironment within tumours. Such a lymphocyte depleted microenvironment may explain the resistance of EGFR overexpressing cancers to tumour therapies, particularly to check-point inhibitor treatments. In this article, we discuss the recent data which support an immune modulatory effect of EGFR signalling and compare these published studies with the most recent data from The Cancer Genome Atlas (TCGA), in this way, dissecting possible underlying mechanisms. We thereby focus our study on how EGFR overexpression may lead to the local activation of TGFβ, and hence to an immune suppressive environment. Consequently, we define a novel concept of how the mitogenic and immune modulatory effects of EGFR overexpression may contribute to tumour resistance to immunotherapy, and how EGFR specific inhibitors could be used best to enhance the efficacy of tumour therapy.

## 1. Introduction

Tumour immunotherapy has made tremendous progress in recent years. Many types of tumours that were considered untreatable only a few years ago currently show promising clinical response rates upon treatment with biologicals, such as monoclonal antibodies targeting so-called immune check-point inhibitors (ICIs), like PD-1/PD-L1 and CTLA-4 [1,2]. These ICIs do not target the tumour itself, but rather they provide relief from inhibitory factors, which prevent immune cells from targeting and eliminating tumour cells [3]. The clinical success of these ICI treatments strongly emphasizes the critical role of immune cells in facilitating tumour regression. Of particular importance are cytotoxic CD8 T-lymphocyte cells, which infiltrate the tumour, recognize tumour specific antigens, and then kill the tumour cells. However, tumour cells can escape from such immunosurveillance; a mechanism which can be mediated in different ways. For instance, tumour cells can create an immunosuppressive tumour microenvironment (TME) via the recruitment of immune-suppressive cells, like CTLA-4 expressing T-regulatory cells (Tregs) or Myeloid-derived Suppressor Cells (MDSC) [4]. Alternatively, tumour cells can up-regulate ligands of T-cell intrinsic check-point inhibitors, such as PD-L1, which blunt CD8 T-cell effector functions [4]. Consequently, treatment of cancer patients with PD-1/PD-L1 and CTLA-4 ICIs can relieve this tumour-intrinsic suppression and allow cytotoxic CD8 T-lymphocytes to re-enter the tumour and clear the tumour cells [5]. Unfortunately, the clinical response to ICI treatment remains limited as clinical responses are observed in only a fraction of patients [2,3,6], with the reasons for a lack of response in the remaining patients remaining largely unresolved.

Strikingly, the clinical response rate to ICI therapies remains unexpectedly poor inpatients with types of epithelial tumours that overexpress either wild-type (wt) or oncogenic alterations of the Epidermal Growth Factor Receptor (EGFR), such as lung, colon, prostate, or pancreas cancers [7,8,9,10]. This is particularly pronounced for EGFR-mutated lung adenocarcinomas [11]. The EGFR functions as a receptor tyrosine kinase and is well-established in activating signal cascades to promote cellular survival and proliferation [12,13,14]. Therefore, it is widely assumed that cancer cells become “addicted” to uncontrolled EGFR-mediated growth and survival signals [15,16]. Support for such a role of EGFR overexpression comes from publications showing that EGFR inhibitors leads to the rapid tumour cell death in cell culture, as well as in mouse models and data from cancer patients [17]. Indeed, targeted small molecule drugs called EGFR tyrosine-kinase inhibitors (EGFR TKI) have been shown to be highly effective in the treatment of lung adenocarcinomas [18,19]. However, the clinical efficacy of EGFR inhibitors still remains limited due to development of tumour resistance; hence, most tumours eventually relapse [11].

Nevertheless, in recent years a second function of mutant and wt EGFR overexpression evolved: one of immune regulation [20]. One line of argumentation was largely based on the efficacy of EGFR inhibitor treatment was largely dependent on the presence of CD4 and CD8 T-cells [21], and that enhanced immune responses were seen following EGFR inhibitor treatment [22,23]. For instance, it was observed that at an early stage following EGFR inhibitor treatment, there was a substantially increased influx of NK cells and lymphocytes, particularly cytotoxic CD8 T cells, as well as dendritic cells, which was accompanied by a decline in Foxp3 expressing Tregs [24,25]. Furthermore, in clinical trials, a lack of correlation between the efficacy of EGFR inhibitor treatment and EGFR expression levels of treated tumours was observed [26,27]. Even tumours with non-detectable EGFR expression responded well to treatment [28]. All these findings strongly suggested that the inhibition of the EGFR not only targeted the tumour cells themselves, but potentially had some influence on tumour-specific immune responses [20].

Furthermore, in a second line of argumentation, it was observed that patients overexpressing mutant forms of the EGFR showed exceptionally low clinical efficacy following the treatment with ICIs, as compared to patients expressing wt EGFR [29,30,31]. Given that EGFR-mediated signalling has been established to upregulate PD-L1 expression on tumour cells [32,33,34,35,36], it had originally been hypothesized that the treatment landscape for such cancers could be revolutionized by CD8 T-cell enhancing immunotherapies like PD-L1 inhibitors [11]. However, for reasons that remain largely unelucidated, EGFR overexpressing tumours instead remain resistant to such treatments [29,30,31,36]. Interestingly, a recent publication revealed a direct, positive correlation between EGFR mutation status and a so called “lymphocyte depletion” phenotype of these tumours, in which there was a pronounced lack of tumor-infiltrating CD8 T-cells [37]. As the absence of tumour infiltrating lymphocytes (TILs), like the cytotoxic CD8 T-cells, is a well-established marker for poor anti-tumour immune responses following ICI treatments [3], these findings suggested that a lack of CD8 T-cells within mutated EGFR overexpressing tumours may explain why such types of tumours showed only poor responses to ICI treatments.

Taken together, these recent studies suggested a rather pronounced immune-modulatory role of EGFR when it was overexpressed, for mutant forms of the EGFR. Given the positive correlation between EGFR mutations and “lymphocyte depletion” phenotype in the recent pan-cancer analysis of The Cancer Genome Atlas (TCGA) [37], we performed a comprehensive TCGA-based analysis of the TME associated with EGFR mutations, with a particular focus on lung adenocarcinomas (LUADs). To this end, we aimed to provide insights into underlying immunotherapy-resistance mechanisms by validating the effect of EGFR mutation types on the immune landscape using a transcriptome-based in silico analysis. Based on the insight gained from this study, we will in the following discuss the findings of different studies and suggest a common mechanism of how the overexpression of mutant forms of the EGFR may contribute to the creation of an immune suppressive tumour microenvironment and the observed “lymphocyte depletion” phenotype.

## 2. The Role of EGFR in Immune Suppression: EGFR Mutations and the Lymphocyte Depletion Phenotype

As mentioned previously, patients suffering from Non-Small Cell Lung Cancer (NSCLC) with EGFR mutations had strikingly poor responses to PD-1 based immunotherapies compared to those with wt EGFR [29,30,31]. This came as a surprise given that numerous in vitro and in vivo studies had previously identified EGFR-mediated signalling as a driver for the upregulation of PD-L1 expression on tumour cells [32,33,34,36] and solid tumours with high PD-L1 expression were expected to be likely responders of PD-L1/PD-1 immunotherapies [3]. Furthermore, immunohistochemistry analyses in multiple studies have highlighted an increased expression of PD-L1 in patients who have NSCLC adenocarcinomas expressing mutated forms of the EGFR [38,39]. Thus, the resistance of tumours overexpressing mutant forms of the EGFR to ICI treatments was unexpected [29,30,31,36]. However, such high correlations between PD-L1 expression and the EGFR mutation status are not uniformly reported across the literature for NSCLC samples including both lung squamous cell carcinoma (SCC) and lung adenocarcinoma (LUAD). Other immunohistochemistry-based studies for instance, identified a lack of significant correlation between EGFR mutant LUADs and high expression levels of PD-L1 [40,41,42]. Such discrepancies can be attributed to several different reasons, including but not limited to small sized biopsies, which may not reflect the heterogeneity of the tumour tissue, as well as differing protocols for tumour sample fixation, antibody staining, scoring methods and interpretation. In addition to such technical limitations, such discrepancies may also arise as a consequence of limited sizes and biases within patient cohorts. Therefore, we assessed PD-L1 expression of lung carcinoma in The Cancer Genome Atlas (TCGA) which provided RNA sequencing data from tumour samples from a larger cohort of patients with NSCLC adenocarcinomas. Nevertheless, our mRNA expression-based analysis showed no significant differences in PD-L1 expression in tumours that either overexpressed mutant forms of the EGFR or had wt EGFR. However, it is important to note that the interpretation of analyses from such mRNA-based studies may have to be treated with caution. Although PD-L1 can be expressed either on the membrane or within the cytoplasm [8]. Only PD-L1 expressed on the membrane is of actual relevance for predicting responses to PD-L1-targeted immunotherapy [8] and an mRNA based assessment does not give any indication about the actual cell surface expression of PD-L1. Nonetheless, although evidence for PD-L1 overexpression in LUAD tumours ultimately remains inconclusive, accumulatively, the discussed data may suggest that PD-L1 expression of tumours with an overexpression of mutant forms of the EGFR might not necessarily be the critical driving force causing the observed resistance to anti-PD-1/PD-L1 treatments.

**Figure 1:**
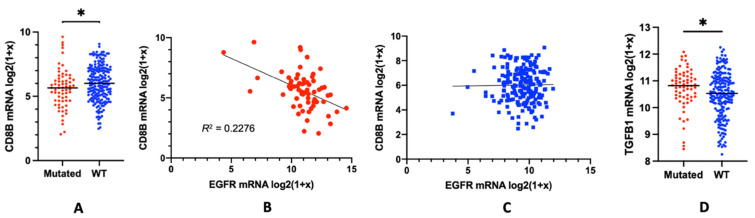
CD8 T-cells frequencies in EGFR expressing NSCLC lung adenocarcinoma (LUAD) tumours. (**A**) Comparison of CD8B mRNA expression (log2(1 + x) transformed data) between LUAD tumours with mutated EGFR (red) (*n* = 66) or wt EGFR (blue) (*n* = 198). Unpaired *t*-test performed to perform statistical group comparison. * Two-tailed *p*-value 0.0424 (**B**) Linear correlation analysis between EGFR mRNA expression (log2(1 + x) transformed data) with CD8B mRNA expression (log2(1 + x) transformed data) in LUAD tumours with mutated EGFR (red) (*n* = 66). Pearson correlation coefficient R^2^ 0.2276 (**C**) Linear correlation analysis between EGFR mRNA expression (log2(1 + x) transformed data) with CD8B mRNA expression (log2(1 + x) transformed data) in LUAD tumours with wt EGFR (blue) (*n* = 198). Pearson correlation coefficient R^2^ 0.0002065 (**D**) Comparison of TGFB1 mRNA expression (log2(1 + x) transformed data) between LUAD tumours with mutated EGFR (red) (*n* = 66) or wt EGFR (blue) (*n* = 198). Mann-Whitney test performed for statistical group comparison. * Two-tailed *p*-value 0.0191. All statistical tests and graphs presented were generated using www.graphpad.com (accessed date 13 Novemeber 2021).

An alternative explanation for the resistance to such ICI treatments might be related to the discussed lack of tumour infiltrating lymphocytes (TILs) in tumours that overexpress mutant forms of the EGFR. Tumours in which high-frequencies of tumour antigen-specific CD8 T-cells infiltrate and reside, are predicted to respond better to ICI treatments than those that lack infiltrates [3]. However, tumours overexpressing mutant forms of the EGFR have previously been associated with a “lymphocyte depletion” phenotype [37]. Such a finding dovetails the results from other studies in the field, which collectively show a lack of CD8 T-cell infiltration in EGFR-mutated tumours [30,43,44]. Given the strong evidence of the association between tumour infiltrating CD8 T-cells with responsiveness to immunotherapies [45,46], we wanted to further explore, in a larger cohort of patients, whether CD8 T-cells were depleted in tumours with EGFR oncogenic mutations. To this end, we measured CD8 expression as a marker of tumour infiltrating CD8 T-cells in NSCLC lung adenocarcinoma (LUAD) samples deposited in the TCGA database, and correlated CD8 with EGFR expression. CD8 can be expressed as a heterodimer between CD8A and CD8B, as well as a homodimer of CD8A only [47]. While the CD8A homodimer is expressed in a wide range of immune cell types, the CD8B containing heterocomplex is expressed exclusively by CD8 T-cells [48]. Therefore, we restricted our analysis on CD8B gene expression as a specific marker for CD8 T-cells and quantified the relative abundance of CD8 T-cells in LUAD tumours with EGFR oncogenic mutations compared to EGFR wild-type (Wt) LUADs. Here, we defined wt LUADs as tumours with diploid copy numbers and no identifiable EGFR mutations or copy number variations. As shown in Figure 1A, we found a significantly diminished expression of CD8B mRNA in tumours overexpressing mutant forms of the EGFR, confirming the described effect of the mutated EGFR expression on reducing the CD8 T-cell infiltration in these tumours [30,37,43,44]. While we found a clear inverse correlation between EGFR expression and CD8 T-cell influx in tumours expressing mutant forms of the EGFR (Figure 1B), we did not find any correlation in those tumours expressing the wt form (Figure 1C). Nevertheless, it is also important to mention that the overall expression of the EGFR was found to be significantly higher in tumours expressing mutant forms than the wt form of the EGFR. Wt EGFR overexpressing tumours, with gene amplification of the wt EGFR gene, were too low in number to be included in this study.

Taken together, in our analysis, we found a direct correlation of the expression of mutant forms of the EGFR and the influx of CD8 T-cells into tumours, directly confirming the findings of other studies [30,37,43,44], which similarly reported that the overexpression of mutant forms of the EGFR leads to an uninflamed, “lymphocyte depletion” phenotype. These findings strongly suggest that mutated-EGFR signalling may have direct tumour-intrinsic immunosuppressive effects on the TME, leading to CD8 T-cell exclusion.

## 3. EGFR Mutations and the Lymphocyte Depletion Phenotype: Potential Mechanisms

One possible underlying mechanism for the lack of response to ICI treatment in cancers expressing mutant forms of the EGFR might be related to an enhanced expansion of Treg populations [49]. Two lines of findings support such an assumption. In vivo studies using mouse models showed that the absence of EGFR and its downstream signaling by genetically deleting the EGFR in tumour cells led to a significantly diminished infiltration of FoxP3 expressing Treg cells in EGFR deficient tumours in comparison to wt EGFR tumours [49]. Such findings suggested that tumours with mutant forms of EGFR leading to constitutively activated EGFR signaling could conversely induce an infiltration of Tregs into the tumour and in this way prevent CD8 T-cell tumor infiltration. Furthermore, pharmacological inhibition of EGFR signalling led to reduced FoxP3 RNA expression in EGFR expressing tumours of treated mice [49]. In addition, a recent study of 19 lung adenocarcinoma patients found high frequencies of Treg infiltration despite low CD8 T-cells in EGFR-mutated lung adenocarcinomas [44], additionally suggesting that EGFR expression may affect Treg expansion within the TME. Therefore, we expanded our TCGA-based analysis on Tregs based on FoxP3 expression as a marker and searched for correlations between FOXP3 expression with EGFR expression. However, we could not find any such correlations between EGFR expression and FoxP3 mRNA expression in tumours expressing either wt or mutated forms of the EGFR. Since FoxP3 is predominantly expressed in Tregs, our data implied that there was no substantial increase of Treg populations within EGFR overexpressing tumours relative to wt EGFR tumours. Previous publications suggested that the increased expression of Treg-recruiting chemokines, such as CCL-22 [44,50] or CCL-5 [51] may have facilitated the influx of CXCR4-expressing Tregs into tumours. Therefore, using the TCGA database, we analyzed the mRNA expression of each of these chemokines in lung adenocarcinoma (LUAD) patients with EGFR oncogenic mutations and compared these gene expression profiles to tumours with wt EGFR. However, our analyses did not show any substantial changes in expression of these chemokines.

In conclusion, these findings suggested that the lack of efficacy of ICI treatment in cancers expressing mutant forms of the EGFR may not depend on an enhanced expansion of Treg populations in EGFR overexpressing tumours. Nevertheless, we would like to emphasize that FoxP3, although expressed on all immunosuppressive Tregs, may not be an ideal marker. In humans, other immune cells, including activated CD4 T-cells, express FoxP3 [52] and some reports even suggest that FoxP3 could be expressed by tumour cells as well [53]. Thus, these EGFR-independent factors leading to FoxP3 expression in the TME may have confounded our regression analysis. Similarly, we only studied the expression of one chemokine per analysis, but we did not analyze the combination of these different chemokines together, which then potentially could have led to an appreciable correlation between EGFR and chemokine expression in those tumours that were resistant to ICI treatment. Therefore, we would like to argue that these data warrant a more sophisticated validation to establish a direct link between chemokine expression and Treg infiltration in the context of lung adenocarcinomas, for instance, by employing well-controlled EGFR-mutated genetically engineered mouse models.

## 4. Alternative Model Proposed: Local TGFβ Activation

**Figure 2:**
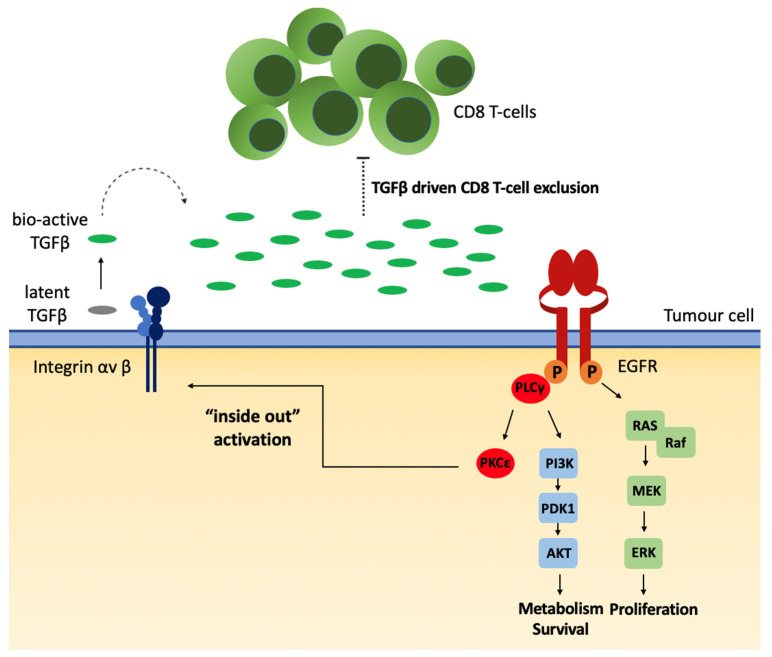
Spontaneous autophosphorylation of EGFR mutants may lead to transforming growth factor beta (TGFβ) activation to induce a lymphocyte depleted tumour microenvironment (TME). Tumour-associated EGFR mutations generate spontaneous autophosphorylation of tyrosine residues that facilitate downstream proliferation and survival signalling pathways. These include the RAS/RAF kinase which mediates the phosphorylation and subsequent activation of the mitogen-activated protein kinase kinase (MEK) cascade which in turn activates the extracellular signal-regulated kinase (ERK). In addition, these downstream signalling pathways also include a sustained activation of the phospholipase C (PLC) signalling pathway. The activation of the PLC signalling pathway induces the phosphoinositide 3-kinase (PI3K)-Akt cascade as well as the activation protein kinase C (PKC), which leads to “inside out” activation of integrin complexes, locally inducing the convertion of latent TGFβ into released bio-active TGFβ. The dashed lines in the illustration represent the proposed mechanism leading to TME immune-suppression. According to the proposed mechanism, the bio-active form of TGFβ released within the TME could potentially exert numerous effects, for instance, leading to the generation of a “lymphocyte depleted” immune suppressive tumour microenvironment via exclusion of tumour killing CD8 T-cells, either directly or indirectly by acting on Tregs.

While our study cannot formally rule out a chemokine mediated influx and subsequent expansion of Treg populations in EGFR overexpressing tumours, the correlation between overexpression of mutated EGFR and CD8 T-cell exclusion (Figure 1) strongly resembled the immune exclusion phenotype driven by the soluble immunomodulatory mediator Transforming Growth Factor beta (TGFβ) [54,55]. TGFβ critically contributes to the local regulation of immune responses and the establishment of a local immune suppressive TME [56]. Importantly, increased TGFβ activation is directly associated with poor responses to PD-L1 immunotherapy due to its association with inducing CD8 T-cell exclusion [55,57]. TGFβ is secreted and stored in a latent form and has to be activated locally [58,59]. Its activation could be mediated via several different ways, for instance by activated integrin-α_V_ chain-containing integrins [60]. Such integrin complexes are ubiquitously expressed, including within tumours [61]. Thus, as an alternative approach, one may suggest that an EGFR-mediated TGFβ activation could be the underlying mechanism leading to local immune suppression and the observed “lymphocyte depletion” phenotype within EGFR overexpressing tumours (Figure 2).

In support of such an assumption, we found a significantly higher expression of TGFβ mRNA in those tumors that overexpressed the EGFR than in those that did not (Figure 1D). Although there is no direct correlation between the expression of latent TGFβ and TGFβ functioning [58,59], we recently have shown that EGFR-mediated signalling can regulate the activation of TGFβ from its latent into its bioactive form [62]. Low-affinity ligands like Amphiregulin activate and selectively induce persistent, tonic EGFR activation of the PLCγ pathway [62,63]. Downstream of a tonic PLCγ signalling, PKC activates and interacts with scaffold protein RACK1 causing intracellular actin cytoskeletal changes and activation of integrin-α_V_ complexes, which subsequently activate TGFβ [64]. Consequently, the overexpression of wt or specific mutated forms of the EGFR can induce a spontaneous, autocatalytic threshold phosphorylation of the EGFR [65,66,67]. This can subsequently induce a sustained, tonic PLCγ-mediated signal and the inside-out activation of integrin-α_V_ complexes leading to TGFβ activation. The assumption that such a tonic PLCγ-mediated signal in tumours overexpressing wt or mutant forms of the EGFR may contribute to the resistance of such tumours to ICI treatment is directly in line with recent findings from the group of Yossi Yarden [36]. However, it is important to note that this group focused entirely on PLCγ-induced PD-L1 signal downstream of the EGFR [36]. In support of our hypothesis that EGFR overexpression may lead to TGFβ activation, preliminary data from our lab suggested an increased release of bioactive TGFβ from tumour cell lines transgenically overexpressing mutant forms of EGFR (data not shown). Within tumours, such a local, consistent activation of TGFβ may then lead to the observed non-inflamed, “lymphocyte depletion” phenotype, which is observed for tumours overexpressing the EGFR.

Strikingly, not all EGFR mutations show the same resistance to PD-L1 checkpoint inhibitors. A number of different mutations are known to alter the tyrosine kinase domain of the EGFR, of which the L858R missense mutation is the most common [65]. Our overall data suggested that the association of CD8 T-cell exclusion likely related to the ability of the EGFR mutations to drive constitutive PLCγ signalling but no MAP-kinase/ERK activation [68]. This might be since most of the common oncogenic missense mutations were located near the tyrosine kinase domain sites where they led to the stabilization of the kinase domain, and thus a latent activation of the receptor [14]. Indeed, in our TCGA-based analysis, we found no significant differences between CD8B expression among LUADs with these common EGFR mutant subtypes, suggesting that for these common EGFR mutation subtypes, the mode of action and the effect on CD8 T-cell infiltration remained the same.

Such an assumption is further supported by the EGFR mutant EGFRvIII, which is frequently found in glioblastoma [69]. Tumours with the EGFRvIII mutation, similar to the common oncogenic EGFR missense mutations, show a constitutive PLCγ signalling but no MAP-kinase/ERK activation could be found at a high level of EGFRvIII expression [69].

Moreover, escape mutants of tumours that become resistant to EGFR inhibitor treatments were often mutants that overexpressed receptor tyrosine kinase, which mainly mediate tonic signals via the PLC signalling pathway, such as IGFR or cMET [17]. These signalling pathways are critical for cell survival, but have a poor mitogenic effect as mitogenic stimuli are mainly mediated via the MAP-kinase/ERK signalling pathway [70]. Hence, it is tempting to speculate that the addictive effect of EGFR overexpression for tumour growth and for tumour resistance might be less based on mitogenic signals mediated via the EGFR than survival and immune-suppressive signal driven by constitutive PLCγ signalling.

## 5. Conclusions

The present study illuminates our understanding of how mutated EGFR correlate with modulated tumour immune responses. The findings from this analysis confirm the association between EGFR oncogenic mutations and CD8 T-cell exclusion. We would like to argue that one possible mechanism contributing to this effect might be the local, EGFR-mediated activation of TGFβ. Hence, TGFβ inhibition strategies may be considered for tumours overexpressing wt or mutant forms of the EGFR. Such treatment strategies may overcome the CD8 T-cell exclusion phenotype and might be useful in cases where EGFR mutant tumours acquire resistance to inhibitors [71]. A more comprehensive understanding of these intricate pathways could thus enable targeted modulation of mutated EGFR tumours to enhance CD8 T-cell driven immunosurveillance and could improve responses to immunotherapies.

Nevertheless, despite the fact that this correlation between the expression of mutant EGFR and CD8 T-cell frequency is rather impressive, we would like to further mention that in addition to the EGFR, many other oncogenic alterations have also been shown to modulate the TME as an immune-evasive mechanism [70]. EGFR oncogenic alterations are concurrently associated with secondary alterations of oncogenic genes including *MYC, MET, TP53, KRAS, PTEN,* and *Wnt*, which may also show immunomodulatory effects [72]. In the TCGA-LUAD cohort used in our analysis, patterns of co-occurrence were noted for alterations in EGFR with *Wnt, PTEN,* and *TP53*, but we were unable to separate the potential CD8 T-cell exclusion effects from those driven by the alterations of these genes. Further studies employing genetically engineered mouse models with EGFR mutations and conditional knockouts for *PTEN* and *Wnt* may be used to elucidate the immunomodulatory role of each gene. By evaluating CD8 T-cell infiltration in the induced tumours, we can confirm the effect of CD8 T-cell exclusion driven by EGFR oncogenic mutation signals. Nonetheless, in most clinical situations, it is important to appreciate that most lung adenocarcinomas with EGFR mutations are likely to have additional co-existing oncogenic alterations. 

Taken together, the identification of the immuno-evasive ability of mutated EGFR by which it drives CD8 T-cell exclusion has the potential to transform the current treatment paradigm. Given the complex and intricate nature of the TME, we hypothesize that the mutated EGFR signalling could be driving tumour cell-autonomous immunosuppression by more than one mechanism. Thus, genomic profiling of circulating tumour DNA from liquid biopsies might be a useful way to identify the oncogenic alterations in sub-clonal altered cells [73]. Accordingly, identification of these specific alterations may allow targeted inhibition to enhance CD8 T-cell responses. Our findings warrant future studies to investigate CD8 T-cell counts in EGFR mutant lung adenocarcinoma responders of such immunotherapies in clinical trials to determine how it affects treatment outcomes. Targeted inhibition of the immunomodulatory signalling pathways associated with the oncogenic alteration can then enrich our armamentarium of cancer therapeutics.

## Data Availability

Publicly available datasets were analyzed in this study. This data can be found here: TCGA database, https://www.cancer.gov/tcga (accessed on 13 November 2021).
